# Supplementary material for: Competition between stochastic neuropeptide signals calibrates the rate of satiation
Source: Res Sq. 2023 Jul 26:rs.3.rs-3185572. Preprint. [Version 1] doi: 10.21203/rs.3.rs-3185572/v1 (PMC10402269; doi:10.21203/rs.3.rs-3185572/v1)
Supplement: Supplement 1 [file NIHPPRS3185572V1-supplement-1.pdf]

Figure S1

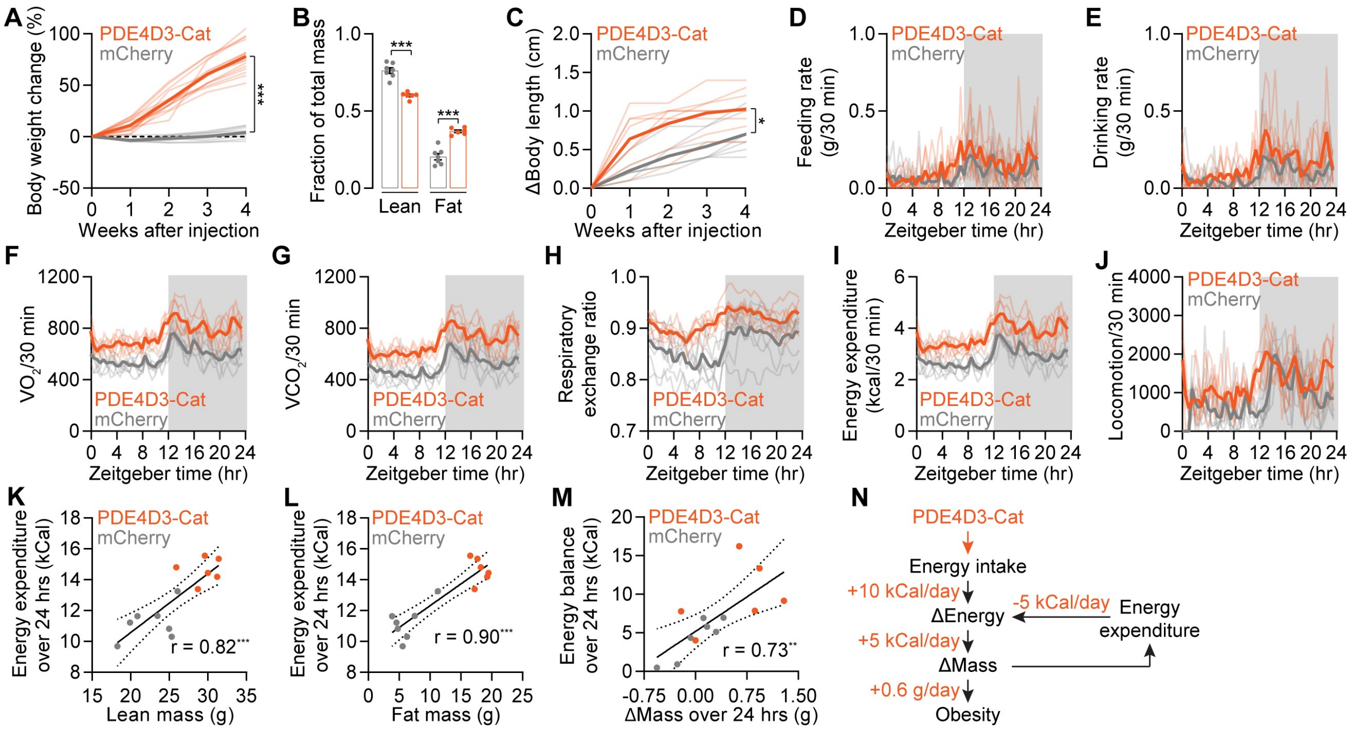

Figure S1. Metabolic changes in mice expressing PDE4D3-Cat in PVH^{MC4R} neurons

(A) AAV expression of PDE4D3-Cat in PVH^{MC4R} neurons in adult mice results in ~80% weight gain in the 4 weeks following surgery (n = 9-15 mice, t-test).

(B) Mice that express PDE4D3-Cat have an elevated contribution of fat mass to their body composition (n = 6-7 mice, One-Way ANOVA).

(C) PDE4D3-Cat expression in PVH^{MC4R} neurons in adult mice results in ~0.5 cm increase in axial length in the 4 weeks following surgery (n = 7-8 mice, t-test).

(D-J) A panel of 24-hr recordings of metabolic parameters in 30-min bins: feeding rate (D), drinking rate (E), VO_2 (F), VCO_2 (G), respiratory exchange ratio (H), energy expenditure (I), locomotor activity (J). n = 6-7 mice. Gray shading: dark cycle.

(K-M) Across individual mice, energy expenditure is well correlated with lean mass (K), fat mass (L), and change in mass (M), in both the presence or absence of PDE4D3-Cat expression. n = 6-7 mice.

(N) Diagram of energy gain: PDE4D3-Cat expression results in increased energy intake (10 kCal/day) that is only partially offset by elevated energy expenditure (5 kCal/day), resulting in a net 5 kCal/day surplus which translates to 0.6 g/day of weight gain. Hence, mice become obese over time.

Figure S2

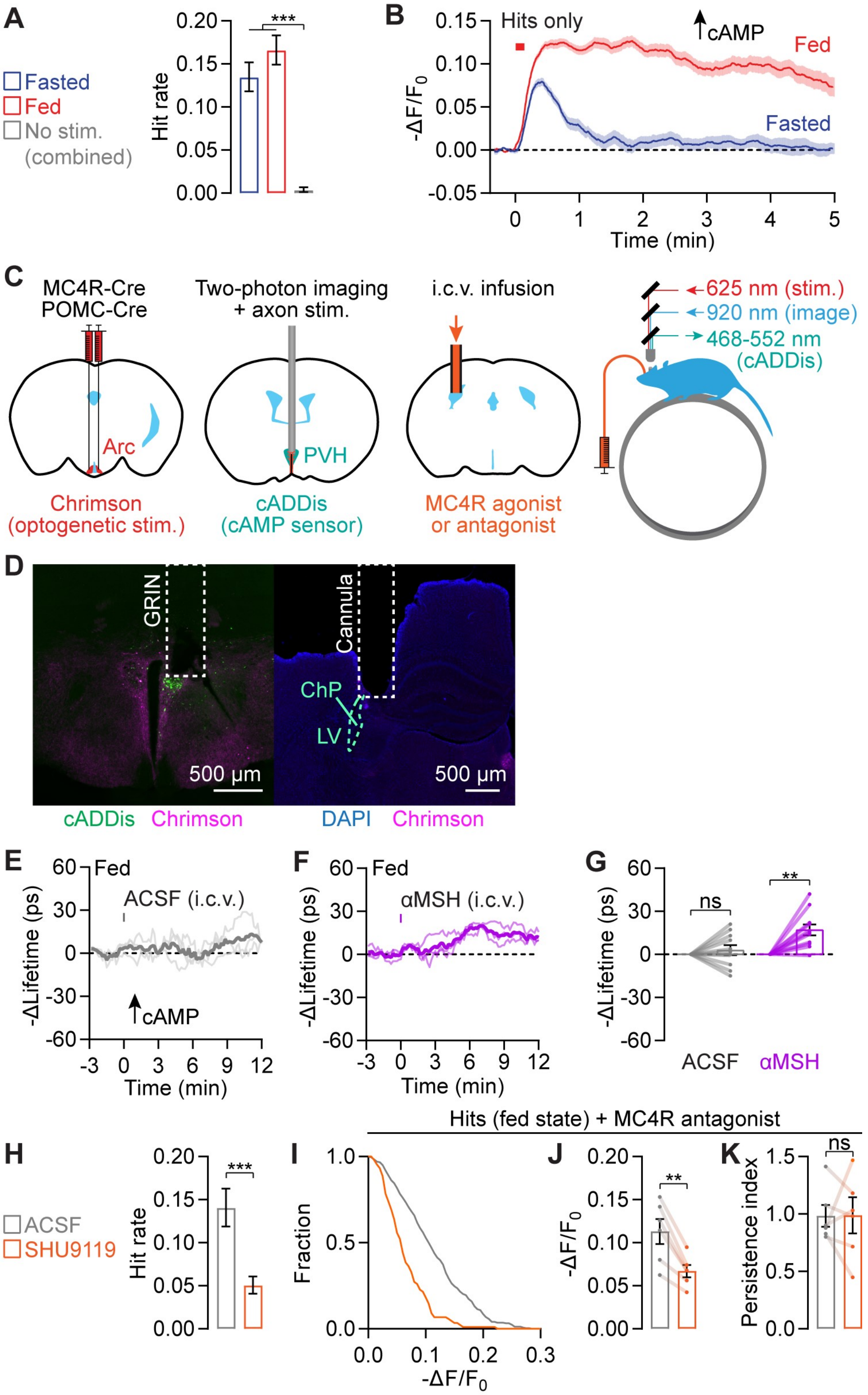
A
Fasted
Fed
No stim.
(combined)
Hit rate
***
B
Hits only
↑cAMP
Fed
Fasted
-ΔF/F0
Time (min)
C
MC4R-Cre
POMC-Cre
Two-photon imaging
+ axon stim.
i.c.v. infusion
625 nm (stim.)
920 nm (image)
468-552 nm
(cADDis)
Arc
PVH
Chrimson
(optogenetic stim.)
cADDis
(cAMP sensor)
MC4R agonist
or antagonist
D
GRIN
Cannula
ChP
LV
500 µm
500 µm
cADDis Chrimson
DAPI Chrimson
E
Fed
ACSF (i.c.v.)
↑cAMP
-ΔLifetime (ps)
Time (min)
F
Fed
αMSH (i.c.v.)
-ΔLifetime (ps)
Time (min)
G
ns
**
-ΔLifetime (ps)
ACSF
αMSH
Hits (fed state) + MC4R antagonist
H
ACSF
SHU9119
Hit rate
***
I
Fraction
-ΔF/F0
J
**
-ΔF/F0
K
ns
Persistence index

Figure S2. Characterizing αMSH signaling to PVH^{MC4R} neurons

(A) Mean hit rates of POMC axon stimulation–induced cAMP increments in PVH^{MC4R} neurons are not different between fasted and fed states. No-stimulation condition in both fasted and fed states shows a low hit rate. n = 1471-3176 trials from 4 mice. Bootstrap comparison of hit rates. Mean ± 95% C.I.

(B) cAMP increments last more than 5 min in fed mice and 1-2 min in fasted mice. n = 211-304 hits from 4 mice.

(C) Complete surgical setup for combining i.c.v. infusion, optogenetic axon stimulation, and cAMP recording through a GRIN lens. Chrimson is expressed in POMC neurons in the arcuate nucleus of the hypothalamus (Arc), and cADDis is virally expressed in PVH^{MC4R} neurons. A GRIN lens is placed above PVH, and a cannula is placed in the posterior lateral ventricle. We chose i.c.v. infusion of peptide antagonist/agonists here and below because many of these drugs do not cross the blood-brain barrier.

(D) Histology of cADDis expression (green) in PVH^{MC4R} neurons and Chrimson-tdTomato expression (red) in POMC axons. In the left panel, dotted lines delineate GRIN lens track. In the right panel, white dotted line delineates the anterior side of the infusion cannula inserted in the lateral ventricle (LV; cyan dotted line) which contains choroid plexus (ChP).

(E-F) Single-mouse fluorescence lifetime traces of cADDis in PVH^{MC4R} neurons in response to infusion of ACSF (E) or 1 nmol αMSH (F). n = 3 mice.

(G) Single field-of-view summary of cAMP increase in PVH^{MC4R} neurons in response to αMSH infusion (n = 11 FOVs from 3 mice).

(H) Pre-infusing MC4R antagonist SHU9119 (1 nmol) reduced the hit rate of POMC stimulation–induced cAMP increments in PVH^{MC4R} neurons (n = 931-1757 trials from 2 mice).

(I) Cumulative distribution of the magnitude of single-trial cAMP increments (measured with cADDis) indicates a reduction in magnitude when SHU9119 was pre-infused before optogenetic stimulation of POMC axons (n = 88-130 hits from 2 mice).

(J-K) Pre-infusing SHU9119 reduced the magnitude of POMC stimulation–induced cAMP increments on hit trials without affecting cAMP persistence (n = 6 FOVs from 2 mice, t-test).

Figure S3

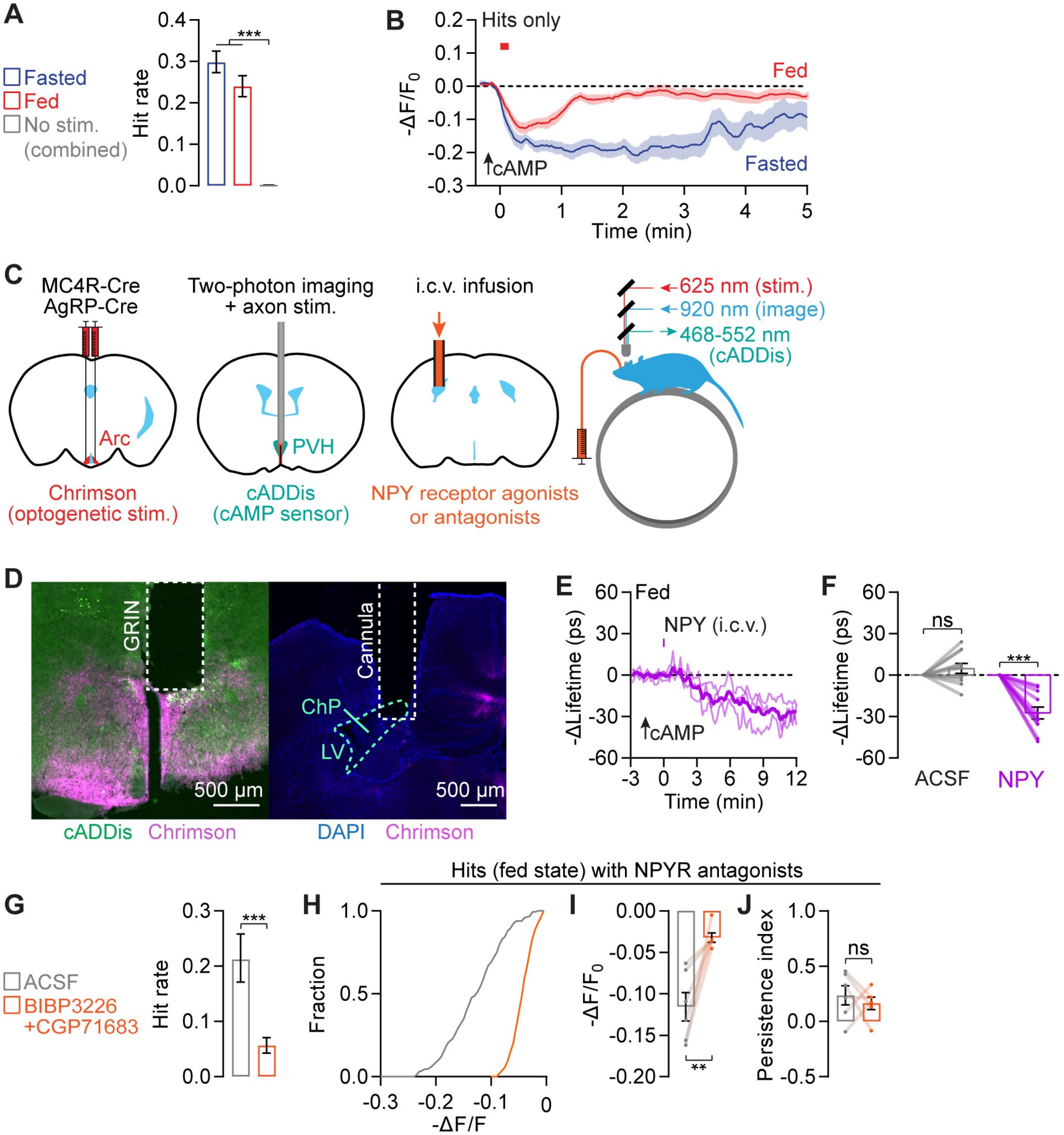

Figure S3. Characterizing NPY signaling to PVH^{MC4R} neurons

(A) Mean hit rates of AgRP axon stimulation–induced cAMP decrements in PVH^{MC4R} neurons are not different between fasted and fed states. No-stimulation condition in both fasted and fed states shows a low hit rate. n = 1093-1928 trials from 4 mice. Bootstrap comparison of hit rates. Mean ± 95% C.I.

(B) cAMP decrements last more than 5 min in fasted mice and 1-2 min in fed mice. n = 262-355 hits from 4 mice.

(C) Complete surgical setup for combining i.c.v. infusion, optogenetic axon stimulation, and cAMP recording through the GRIN lens. Chrimson is virally expressed in AgRP neurons in the arcuate, and

cADDis is virally expressed in PVH^{MC4R} neurons. A GRIN lens is placed above PVH, and a cannula is placed in the posterior lateral ventricle.

(D) Histology of cADDis expression (green) in PVH^{MC4R} neurons and Chrimson-tdTomato expression (red) in AgRP axons. In the left panel, dotted lines delineate the GRIN lens track. In the right panel, white dotted line delineates the anterior side of the infusion cannula inserted in the lateral ventricle (LV; cyan dotted line) which contains choroid plexus (ChP).

(E) Single-mouse fluorescence lifetime traces of cADDis in PVH^{MC4R} neurons in response to infusion of 0.5 nmol NPY. n = 3 mice.

(F) Single field-of-view summary of decrease in cAMP in PVH^{MC4R} neurons in response to infusion of 0.5 nmol NPY (n = 11 FOVs from 3 mice).

(G) Pre-infusing NPY1R antagonist BIBP3226 (10 nmol) together with NPY5R antagonist CGP71683 (10 nmol) reduced the hit rate of AgRP stimulation–induced cAMP decrements in PVH^{MC4R} neurons (n = 680-909 trials from 2 mice).

(H) Cumulative distribution of magnitudes of single-trial cAMP decrements (measured with cADDis) indicates a decrease in magnitudes when BIBP3226 and CGP71683 were pre-infused before optogenetic stimulations (n = 55-144 hits from 2 mice).

(I-J) Pre-infusing BIBP3226 and CGP71683 reduced the magnitude of AgRP stimulation–induced cAMP decrements without affecting the persistence of these cAMP decrements (n = 6 FOVs from 2 mice, t-test).

Figure S4

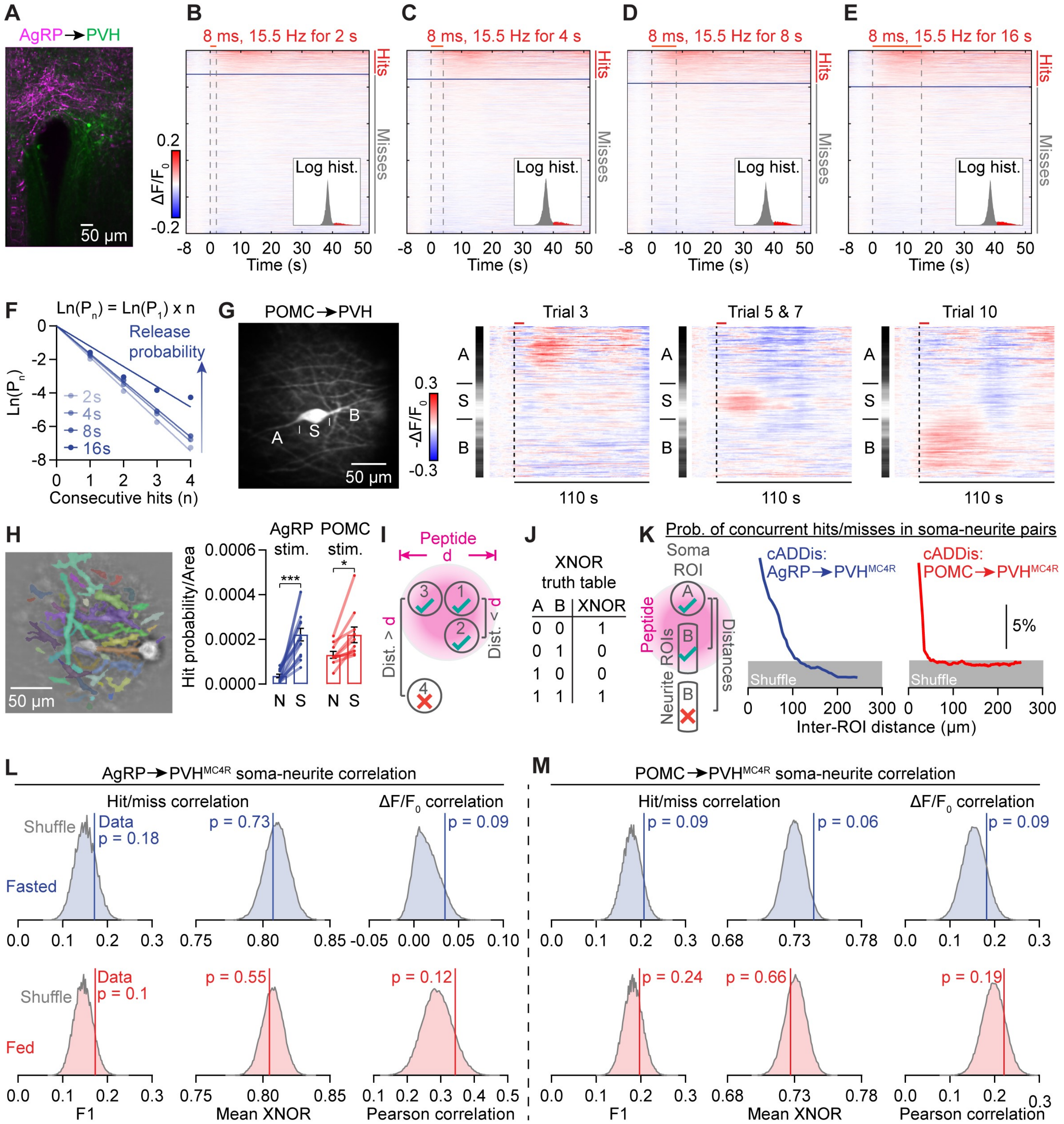

Figure S4. Stochastic neuropeptide release triggers spatially discrete cAMP signals

(A) Example field view of npyLight expression in PVH (green) and Chrimson-tdTomato expression in AgRP axons (red).

(B-E) Summary heatmaps of single-trial npyLight signals in response to 2 s (B), 4 s (C), 8 s (D), or 16 s (E) of Chrimson photostimulation (n = 16439-19969, trials from 7 mice). Trials are sorted by peak intensity.

Inset: distribution of peak intensities, color-coded red for hits and gray for misses, with x-axis on a log scale.

(F) The hit rates of AgRP stimulation–induced NPY signals (measured with npyLight) are mostly well described using the dice model, with a modest, sublinear increase in probability of release with increasing stimulation duration (n = 16439-19969 trials from 7 mice).

(G) In an example neuron with a clearly visible soma (S) and two associated neurites (A and B), POMC stimulation–induced cAMP increments are spatially localized and occur on different trials for the soma and for each neurite.

(H) Left: example neurite segmentation by Cellpose 2.0 nuclear model that is retrained by manual neurite segmentation (see Methods). Right: During both AgRP axon stimulation and POMC axon stimulation, the hit rate per area is non-zero in neurites (N) but is lower in neurites than soma (S; n = 10-13 FOVs from 8 mice, one-way ANOVA).

(I) Model: during spatially restricted peptide release, ROIs that are closer to each other than the impact diameter are more likely to receive the same peptide signal than ROIs that are further apart.

(J) The truth table of exclusive not-or (XNOR), a metric of concurrence between pairs of binary events, A and B. When cAMP responses in two regions-of-interest concur on a given trial (e.g. both exhibit a hit or both exhibit a miss), the XNOR value equals one.

(K) Probability of concurrent hits or misses between soma-neurite pairs drops as the distance between the two ROIs increases. The distance beyond which concurrence of cAMP responses drops to chance levels is ~100 μm during both AgRP stimulation (left) and POMC stimulation (right).

(L) During AgRP axon stimulation in both fasted (blue) and fed (red) states, the soma-neurite signals are consistently decorrelated from each other. This is evident using F1-score analysis of binary hit/miss data, XNOR analysis of binary hit/miss data, or Pearson correlation of continuous $\Delta F/F_0$ data. Vertical lines are experimental data, and shaded area shows the bootstrapped distribution (100,000 iterations, with replacement). P-value indicates two-tailed probability that actual concurrence estimate (F1, XNOR or Pearson correlation) falls outside the mean of shuffled values.

(M) Same as L, but for POMC axon stimulation.

Figure S5

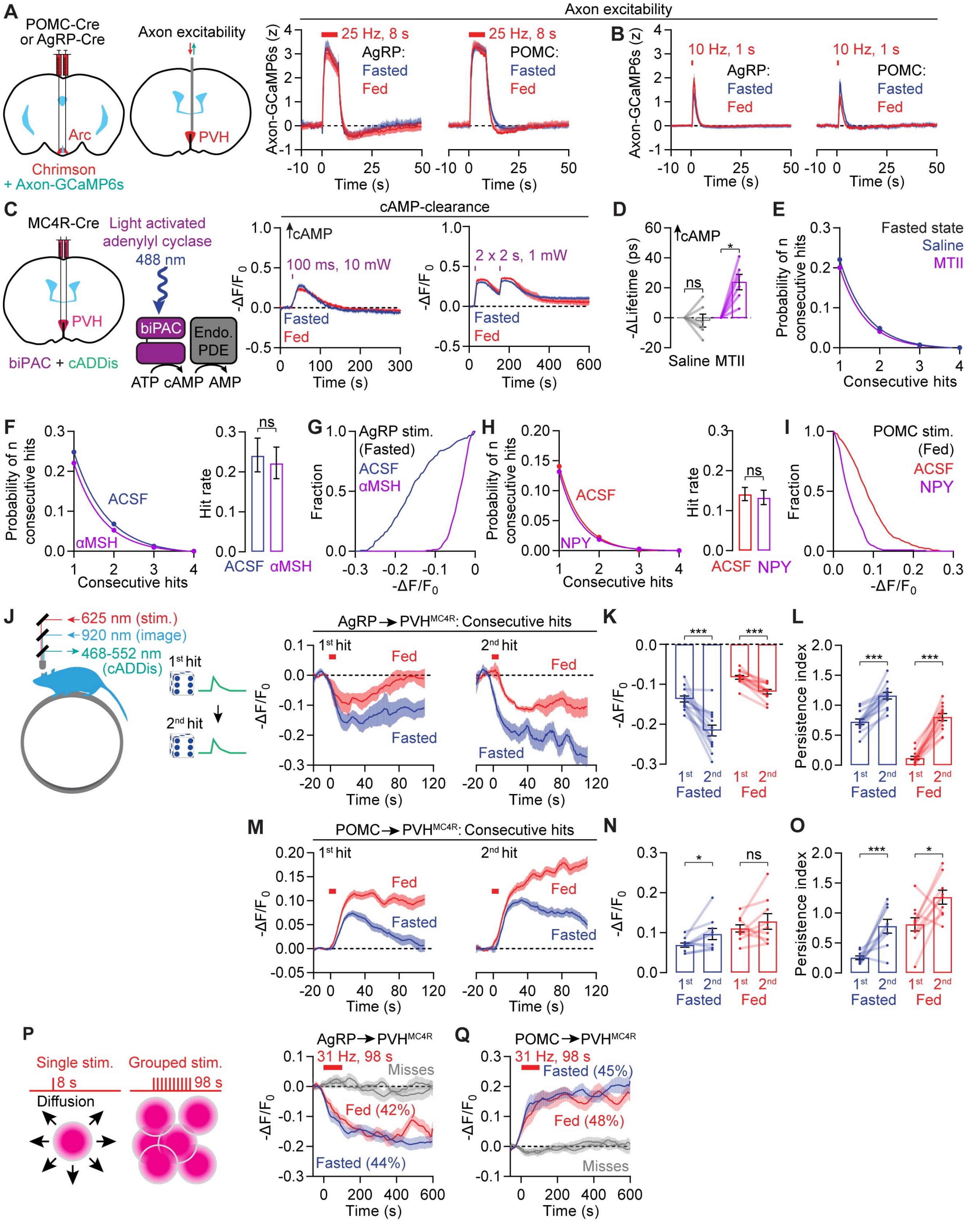

2038

Figure S5. Additional evidence for the neuropeptide competition hypothesis.

(A) To measure potential axon excitability differences across states, which could lead to differences in peptide release, we first co-expressed Chrimson and Axon-GCaMP6s in AgRP neurons and, in separate experiments, in POMC neurons. In both cases, we implanted an optic fiber in PVH to photostimulate Chrimson-expressing axons and to record the resulting calcium transients in these same axons. Axon calcium transients evoked by 25-Hz, 8-s Chrimson stimulation in AgRP (left) and POMC axons (right) were similar in fasted and fed states (n = 9 mice).

(B) Brief 10 Hz, 1 s Chrimson stimulation of AgRP axons triggers stronger calcium transients in the fed state (when NPY signaling is weaker). The same photostimulation of POMC axons triggers stronger calcium transients in the fasted state (when αMSH signaling is weaker). These differences are presumably due to lower baseline activity of AgRP axons and POMC axons in the fed and fasted state, respectively. n = 9 mice. Note that these differences argue against a major contribution of presynaptic excitability to the state-dependent differences in cAMP responses: AgRP stimulation drives weaker PVH^{MC4R} cAMP responses in the fed state, despite the slightly *stronger* 1-s stimulation-evoked AgRP axon calcium signals in the fed state. Similarly, POMC stimulation drives weaker PVH^{MC4R} cAMP responses in the fasted state, despite the slightly stronger 1-s stimulation-evoked POMC axon calcium signals in the fasted state.

(C-D) We used blue-light activation of the optogenetic adenylyl cyclase, biPAC, to bypass endogenous peptide receptor signaling and directly produce cAMP in PVH^{MC4R} neurons in slices, while monitoring cAMP dynamics with the sensor cADDis. cAMP produced by biPAC photostimulation (1x 100 ms or 2x 2 s) in PVH^{MC4R} neurons is cleared by endogenous PDEs at similar rates in fasted and fed states, arguing against state-dependent cAMP degradation (n = 8 slices from 4 mice).

(D) MTII injection (3 mg/kg, i.p.) elevates cAMP in PVH^{MC4R} neurons using two-photon fluorescence lifetime imaging *in vivo* (n = 6 FOVs from 3 mice, one-way ANOVA).

(E) MTII pre-injection does not change the hit rate of AgRP stimulation–induced cAMP decrements (n = 473-517 trials from 3 mice).

(F-G) αMSH pre-injection does not change the hit rate of AgRP stimulation–induced cAMP decrements (F) but reduces hit magnitudes (G). n = 391-473 trials from 2 mice, bootstrap comparison of hit rates.

(H-I) NPY pre-infusion does not change the hit rate of POMC stimulation–induced cAMP increments (H) but reduces hit magnitudes (I). n = 1311-1667 trials from 2 mice, bootstrap comparison of hit rates.

(J-L) When analyzing two consecutive hits of AgRP stimulation–induced cAMP decrements, the second decrement is larger than the first (n = 13 FOVs from 4 mice). Amplitudes are calculated as $-\Delta F/F_0$ means in the 20-40 s window following stimulation onset. Baselines are calculated separately for first and second hits to prevent lingering elevation from the first hit from contributing to the calculations of the second.

(M-O) The same as J-L but for POMC stimulation–induced cAMP increments (n = 10 FOVs from 4 mice).

(P-Q) To more directly manipulate the total amount of peptide released in a local region of PVH, we presented groups of ten 8-s photostimulations of AgRP and POMC axons with a much shorter inter-stimulation interval (2 s instead of 52 s; other experimental parameters were not modified). The shorter inter-stimulation intervals within each 98-s stimulation sequence should decrease the degree to which peptides released during each 8-s stimulation diffuse away or are broken down by peptidases (Xiong *et al.*, 2022; Turner *et al.*, 1985) between trials, resulting in greater accumulation of extracellular neuropeptide levels that could overcome endogenous competition from opposing neuropeptides (illustrated in P). Consistent with this prediction, these 98-s groups of AgRP or POMC axon stimulations drove cAMP decrements (P; 42-44% hit rate per group) and increments (Q; 45-48% hit rate) that were

long-lasting (>8 min after the last pulse) and insensitive to hunger state. P: n = 112-167 trials from 4 mice, Q: n = 97-139 trials from 4 mice.

Figure S6

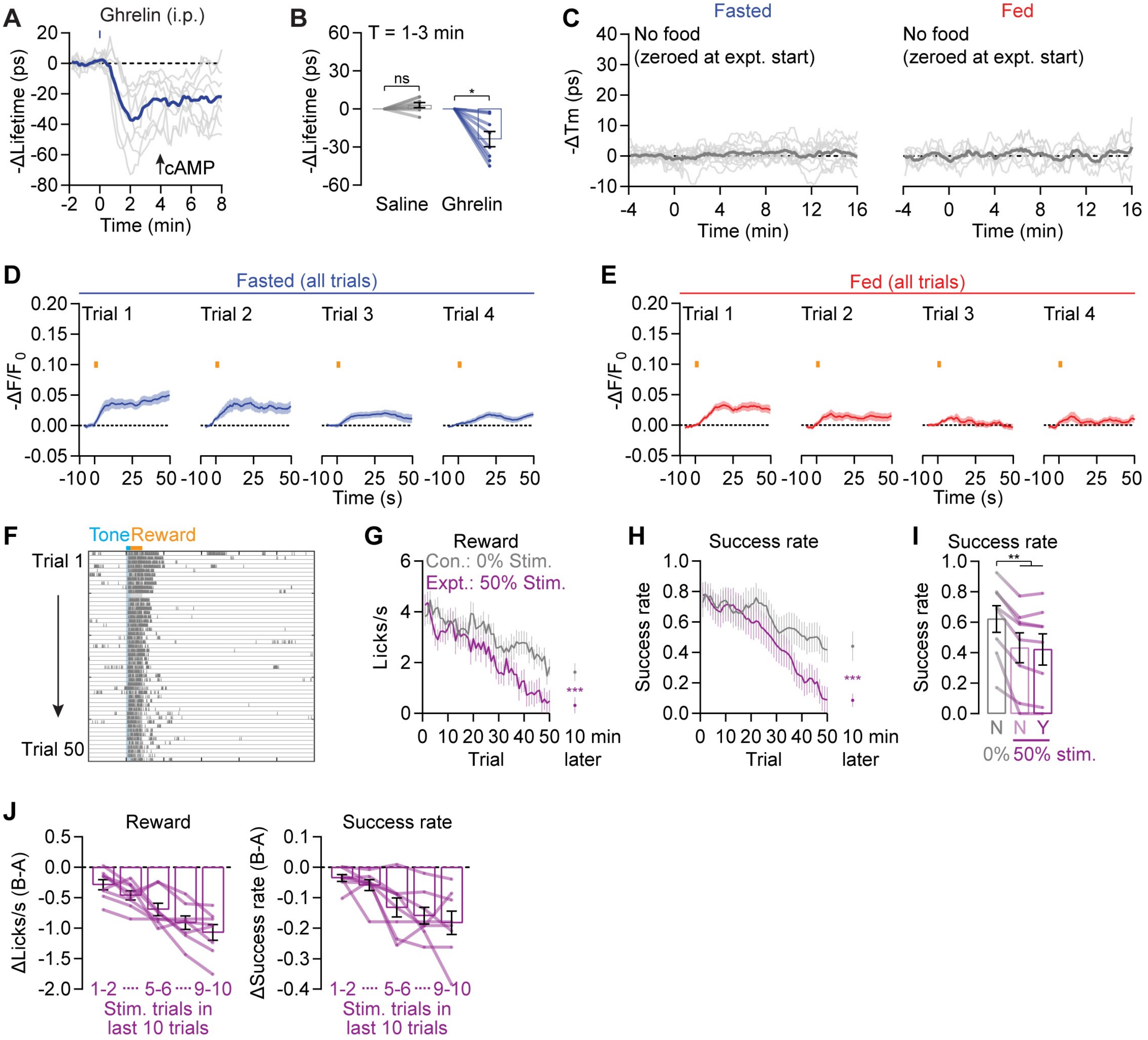

Figure S6. cAMP in PVH^{MC4R} neurons gradually promotes satiation.

(A-B) Injection of ghrelin (2.5 mg/kg, i.p.), which stimulates AgRP neurons and inhibits POMC neurons, induces a robust decrease in cAMP in PVH^{MC4R} neurons in fed mice.

(C) cAMP levels are stable in PVH^{MC4R} neurons in the absence of feeding (n = 8 FOVs from 3 mice), arguing against any non-stationarities due to elapsed time within a session.

(D-E) Trial-average cAMP increments in the fasted (D) and fed states (E) in the first four trials. D: n = 561 trials from 6 mice, E: 646 trials.

(F) In an assay where fasted mice lick during a tone (blue) to obtain reward (orange, milkshake), well-trained food-restricted mice start licking during the tone but the lick rate gradually decreases over 50 trials.

(G-H) In experimental sessions (purple), in which biPAC stimulation was delivered in 50% of the trials, lick rates (G) and success rates (H) drop off faster than in control sessions (gray), and do not recover after 10 min without additional cues or reward deliveries. n = 8 mice, t-test.

(I) Within the experimental session, no difference in success rate (rate at which the mouse exhibited licking following the tone but prior to the reward delivery) was observed between stimulation trials (purple, ‘Y’) and no-stimulation trials (purple, ‘N’). n = 8 mice, one-way ANOVA.

(J) Over a window of 10 trials, the decrease in lick rate during reward (left), and success rate of correct licking following the cue (right) scale with the total number of biPAC stimulations delivered across the last ten trials (n = 8 mice). Together with panel I, this suggests that the effects of cAMP increments are gradual (i.e., they do not affect same-trial performance) and cumulative across minutes.

Figure S7

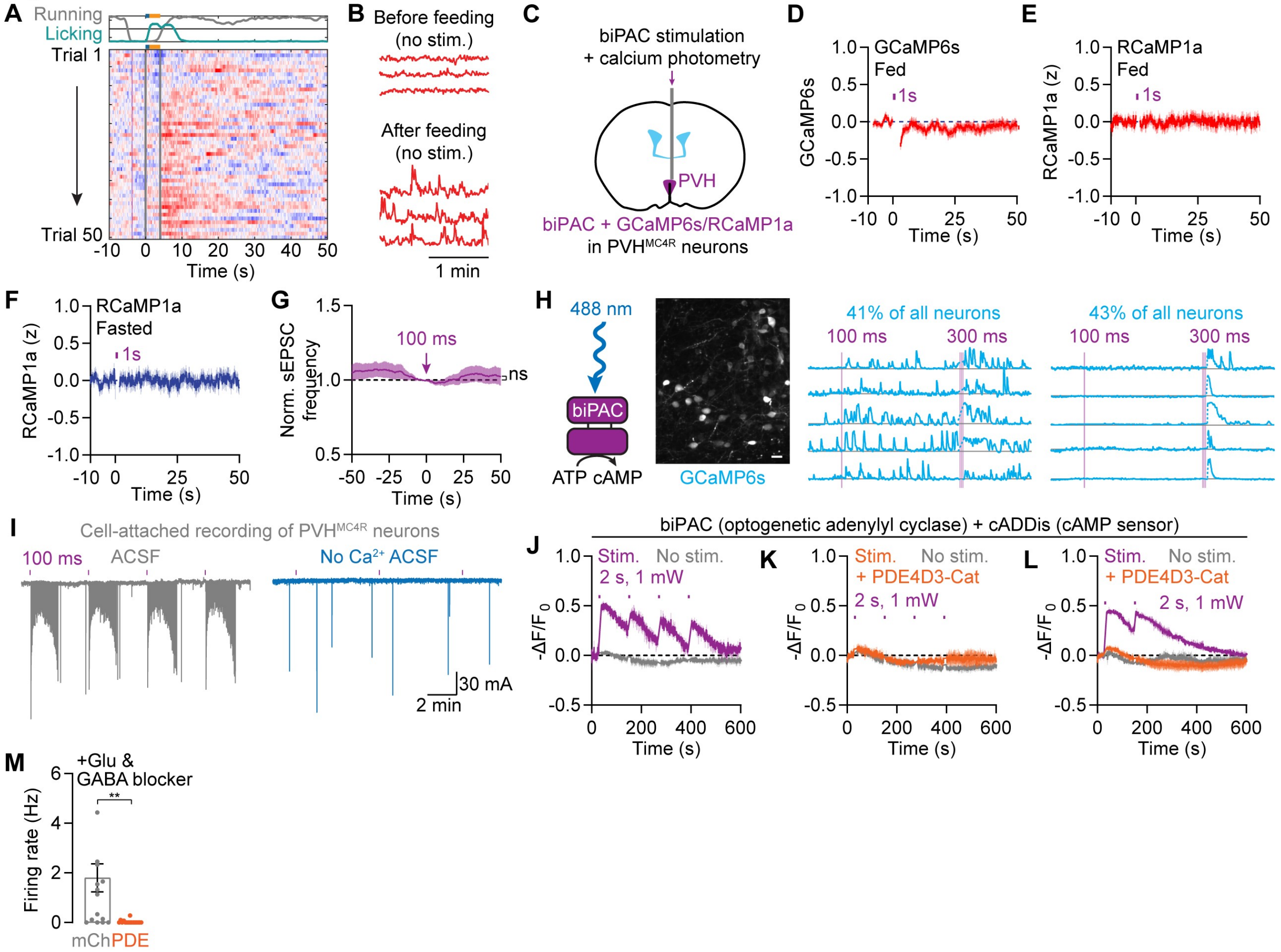

Figure S7. cAMP potentiates excitatory inputs to PVH^{MC4R} neurons.

(A) An example session from a fasted mouse that licks during the cue (blue) to obtain reward (orange). Panel shows a heatmap of RCaMP1a photometry signals (each row is a trial). There is a delayed increase in calcium activity during each trial (which peaks ~4s after cue onset, and several seconds after consumption onset), potentially reflecting ingestion-related gastrointestinal signals. This increase in activity takes ~10 trials to develop.

(B) Photometry recording of spontaneous bulk calcium activity after a session suggests increases in ongoing PVH^{MC4R} calcium activity after feeding.

(C-F) In mice co-expressing biPAC and a calcium sensor (GCaMP6s or RCamp1a) in PVH^{MC4R} neurons, briefly stimulating biPAC through a fiber does not result in noticeable calcium transients in either fed mice (D and E) or fasted mice (F). We tested RCaMP1a, a green-light sensitive calcium sensor, to avoid biPAC activation by photometry light. Blanking (2-4 s) in traces is done to remove temporary photobleaching due to optogenetic stimulation. n = 8 mice per panel.

(G) Brief 100-ms biPAC stimulation did not increase the frequency of spontaneous excitatory inputs, thereby sensitizing PVH^{MC4R} neurons to excitatory inputs. n = 16 cells from 3 fed mice.

(H) In acute brain slices, brief biPAC activation (100 ms or 300 ms, 10 mW) resulted in changes in calcium activity that can be described in two categories: 41% of PVH^{MC4R} neurons showed a persistent elevation in calcium activity, while a different 43% showed relatively transient (~100 s) increases in calcium activity (n = 5 slices from 2 fed mice). Because the transient activation was not seen *in vivo* (see Figure S7C-S7F), we did not pursue it further.

(I) Example cell-attached recording shows elevated firing rate (negative deflections) for ~2 min after each brief biPAC stimulation pulse (100 ms). The acute neuronal activation by biPAC stimulation was not seen *in vivo* (see Figure S7C-S7F). Such a difference between slice and *in vivo* results could be due to lower extracellular calcium concentration *in vivo,* as the excitability effects in slice depended on extracellular calcium (right).

(J-L) In acute brain slices, brief biPAC activation (2 s, 1 mW) induces cAMP increments in PVH^{MC4R} neurons that gradually decay back to baseline. Co-expressing PDE4D3-Cat completely blocks biPAC-induced cAMP transients and therefore should also reduce feeding-related cAMP increments. Traces in L allow for longer time to visualize cAMP decay. n = 3-5 slices from 3 mice total.

(M) In slices with both glutamate and GABA blockers, PDE4D3-Cat-expressing cells almost never show spontaneous spikes (n = 6-7 cells from 4 fed mice total).

Supplementary Files

This is a list of supplementary files associated with this preprint. Click to download.

- MC4RpaperTableS1.pdf
